# A comparative assessment of antiproliferative properties of resveratrol and ethanol leaf extract of *Anogeissus leiocarpus* (DC) Guill and Perr against HepG2 hepatocarcinoma cells

**DOI:** 10.1186/s12906-017-1873-2

**Published:** 2017-08-02

**Authors:** Jeremiah Olorunjuwon Olugbami, Robert Damoiseaux, Bryan France, Esther Modupe Onibiyo, Michael Adedapo Gbadegesin, Shivani Sharma, James Kazimierz Gimzewski, Oyeronke Adunni Odunola

**Affiliations:** 1 Department of Biochemistry, Cancer Research and Molecular Biology (CRMB) Laboratories, University of Ibadan, Ibadan, Oyo, Nigeria; 2 Department of Chemistry and Biochemistry, University of California, Los Angeles, California USA; 3 Nano and Pico Characterisation (NPC) Core Facility, California NanoSystems Institute (CNSI), University of California, Los Angeles, California USA; 4 Molecular Screening and Shared Resources (MSSR) Core Facility, California NanoSystems Institute (CNSI), University of California, Los Angeles, California USA; 5 Department of Molecular and Medicinal Pharmacology, University of California, Los Angeles, California USA; 6 Department of Chemical Sciences, Ajayi Crowther University, Oyo, Oyo Nigeria; 7 International Center for Materials Nanoarchitectonics Satellite (MANA), National Institute for Materials Science (NIMS), Tsukuba, Japan

**Keywords:** Free radicals, Resveratrol, *Anogeissus leiocarpus*, HepG2 cells, Cell death, Cell proliferation, Morphological changes

## Abstract

**Background:**

Epidemiological and experimental evidences have shown cancer as a leading cause of death worldwide. Although the folklore use of plants as a reliable source of health-restoring principles is well-documented, the search for more of such plants that are active against diseases, such as cancer, continues. We report here a laboratory-based evidence of the relevance of an ethanol leaf extract of *Anogeissus leiocarpus* (A2L) in comparison with resveratrol, a natural polyphenol, in cancer therapy.

**Methods:**

The quantitative assessment of flavonoid and phenolic contents involved quercetin and gallic acid as standards, respectively were determined using spectrophotometry. Cytotoxicity was determined fluorometrically using propidium-iodide-staining method. Antioxidant status, adenosine triphosphate (ATP) levels, caspase activities and mitochondrial integrity were assessed using fluorometry/luminometry.

**Results:**

The antioxidant assay demonstrated that A2L possesses a strong antioxidant capacity as compared with the reference compounds, ascorbic acid and butylated hydroxytoluene. This is further buttressed by the significantly high level of phenolics obtained in the quantitative assessment of the extract. A 72-h post-treatment examination indicated that both A2L and resveratrol modulate the proliferation of HepG2 liver carcinoma cells in a time- and concentration-dependent manner. Determination of the total nuclei area, propidium-iodide negative and positive nuclei areas all further buttress the modulation of cell proliferation by A2L and resveratrol with the indication that the observed cell death is due to apoptosis and necrosis at lower and higher concentrations of treatments respectively. At lower concentrations (0.39–3.13 μg/mL), resveratrol possesses higher tendencies to activate caspases 3 and 7. Bioenergetically, both resveratrol and A2L do not adversely affect the cells at lower concentrations (0.39–6.25 μg/mL for resveratrol and 12.5–100.0 μg/mL for A2L) except at higher concentrations (12.5–25.0 μg/mL for resveratrol and 200–800 μg/mL for A2L) which are more pronounced in A2L-treated cells. Furthermore, the antioxidant status of HepG2 cells is not perturbed by resveratrol as compared with A2L. Assessment of 24-h post-treatment mitochondrial function shows that resveratrol is not mitotoxic as compared with A2L which exhibits mitotoxicity at its highest concentration.

**Conclusions:**

Taken together, findings from this study showed that A2L possesses strong antiproliferative activity and its prospect in the management of hepatocellular carcinoma deserves further investigation.

## Background

The current statistics reveal that cancer is now a leading cause of death worldwide, and liver cancer is one of the deadliest forms. Liver cancer has been reported to be the second most common cause of death from cancer globally after lung cancer [1]. Increasingly new cases of liver cancer were diagnosed, from 626,162 in 2002 to 782,451 in 2012. This figure is projected to increase to 1,341,344 cases by 2035 [1].

There has been an increased search for anticancer medicinal plant products based on the fact that surgical and traditional therapeutic approaches (chemotherapy and radiation) are, at present, unable to control most cancer types [2]. Furthermore, considering the concomitant toxic effects associated with the use of synthetic chemicals in cancer therapy, scientific research efforts are being geared toward naturally-occurring plant sources which are therapeutically effective, culturally acceptable and economically accessible [3].

*Anogeissus leiocarpus* (DC) Guill and Perr (English/common name: Axlewood tree; Yoruba name: Ayin) is one of the medicinal plants contained in the Nigeria’s diverse flora (Dalziel, 1937). It has varied applications in Nigerian traditional medicine mostly in the treatment of respiratory diseases, ascaricide, gonorrhoea, general body pain, blood clots. [4]. The result of an oral acute toxicity study revealed no death with doses up to 3200 mg/kg body weight in an animal model. Furthermore, histopathological lesions were not observed in all the organs except the lungs, which showed congestion, oedema and bronchitis. These results suggested that the leaf extract of *A. leiocarpus* could be used with some degree of safety especially by oral route [5].

Resveratrol (3,5,4′-Trihydroxystilbene) is a natural polyphenol which was isolated from the root of *Veratrum grandiflorum* by Takaoka with its chemical structure characterised in 1940 [6]. Studies have shown that resveratrol can exert antitumour effects at the stages of cancer initiation, promotion and progression [7]. Moreover, resveratrol has also received considerable attention due to its potential chemopreventive and chemotherapeutic properties [8]. This study, therefore, evaluated the in vitro cell modulatory properties of ethanol leaf extract of *A. leiocarpus* (A2L) as compared with resveratrol in HepG2 liver carcinoma cell line.

## Methods

### Collection of leaves of *A. leiocarpus* and preparation of its ethanol extract

*A. leiocarpus* leaves were collected from Lokoja, Nigeria and authenticated at the Department of Botany, University of Ibadan with the reference number: UIH 22402. The sample leaves were washed with distilled water (dH_2_O) to remove any contaminants, air-dried under shade until it attained a constant weight, grinded to powder, sieved, packed into polythene bags and stored at 4 °C. Four hundred grams (400 g) of the powered plant part was macerated in 70% ethanol (1600 mL) for 72 h with intermittent stirring/shaking [9]. At the end of the extraction, the extract was filtered using Whatman filter paper No.1 (Whatman Ltd., England) to remove all unextractable matters, including cellular materials and other constituents that are insoluble in the extraction solvent. The filtrate was concentrated using a rotary evaporator (RE-52A, Shanghai Ya Rong Biochemistry Instrument Factory, Shanghai) under reduced pressure (in order to speed up the process) at 40 °C and stored at 4 °C until when needed. The percentage yield of the extraction was 14.17% *w*/w.

### Determination of total phenolic content

The amount of total phenolics in the plant extract was determined with the Folin-Ciocalteau reagent using the method of Spanos and Wrolstad [10] with some modifications an in Olugbami et al. [11]. Briefly, 2.5 mL of 1/10 dilution of Folin-Ciocalteau’s reagent and 2 mL of Na_2_CO_3_ (7.5% *w*/*v*) were added to 0.50 mL of the extract (800 μg/mL) and incubated at 45 °C for 15 mins. The absorbance of was measured at 765 nm using UV/VIS spectrometer T70. Results are expressed as micrograms of gallic acid equivalents per milligram of dry weight (μg GAE/mg) of extract.

### Determination of total flavonoid content

This was carried out by using the method described by Nickavar and colleagues [12]. Briefly, 2.5 mL of the extract (800 μg/mL) was mixed with 2.5 mL AlCl_3_ reagent in 90% ethanol and allowed to stand for 40 mins at room temperature. After that, the absorbance of the mixture was measured at 415 nm using UV/VIS spectrometer T70. The blank was made up of 2.5 mL of 90% ethanol plus sample solution (2.5 mL). The TFC which is expressed as micrograms of quercetin equivalents per milligram (μg QE/mg) of extract was determined on the basis of the linear calibration curve of quercetin (absorbance versus quercetin concentration).

### Cell line and reagents

HepG2 (human liver carcinoma) cell line used in this study was generously provided by Dr. Samuel French (Dept. of Pathology and Laboratory Medicine, University of California, Los Angeles, CA, USA). The cells were grown in Dulbecco’s Modified Eagle’s Medium [DMEM] (Sigma-Aldrich, MO, USA) supplemented with 10% (*v*/v) fetal bovine serum (Sigma Aldrich, MO, USA), 1% L-glutamine (Sigma Aldrich, MO, USA) and 1% penicillin/streptomycin (Sigma Aldrich, MO, USA). The cells were cultured in a water-jacketed incubator (Forma Scientific, MO, USA) at 37 °C, 5% CO_2_, 95% air with the renewal of medium being every 2–3 days. Resveratrol, dimethyl sulphoxide (DMSO), 0.05% trypsin, 0.4% trypan blue, and propidium iodide (PI) were purchased from Sigma Aldrich, MO, USA. Hoechst 33,342 was from Invitrogen, USA while GSH/GSSG-Glo™, Caspase-Glo 3/7, and Mitochondrial ToxGlo™ Assay reagents were obtained from Promega Corporation, WI, USA.

### Cell counting, image acquisition and analysis

Cells were harvested and re-suspended in medium to ensure a single cell suspension and then counted on a haemocytometer based on trypan blue dye exclusion method (0.4% trypan blue; Sigma-Aldrich, St Louis, MO) by adding 10 μL of trypan blue solution to 10 μL of cell suspension (1:1 *v*/v). ImageXpress® Micro Widefield High Content Screening System and its associated MetaXpress® High Content Image Acquisition and Analysis Software interface were utilised for image acquisition and analysis. After dye addition and incubation (1 h), images were captured with two software predetermined excitation/emission filter sets; 380/535 nm for Hoechst and 555/645 nm for PI. Transmitted light and fluorescence images from each well were acquired using a 20× and 4× dry objective lenses respectively. Under these settings and by plating HepG2 cells at the predetermined optimum cell densities indicated above, the system was able to capture from ~100 to 400 cells per well and the images were subsequently analysed using the MetaXpress® Analysis Software. To define nuclei as individual units or regions of interest (ROIs), pre-processing filters and intensity thresholds were applied for image segmentation. Segmented images were subjected to data classification by the use of the MetaXpress® Analysis Software. The percentage of dead cells was calculated from the total number of ROIs per well. Cell nuclei emitting fluorescence signal from both Hoechst and PI (fluorescence co-localisation) were considered as dead cells, while cells emitting only Hoechst signal were counted as live cells.

### Cytotoxicity determination

Resveratrol (100 mg) was prepared as 20 mg/mL by adding 5 mL of 100% DMSO and then sonicated to ensure total dissolution of the solute. While the extract was prepared in 0.5% DMSO, sonicated and centrifuged at 13500 rpm with the supernatant preserved for subsequent use. For the assays, resveratrol and extract were further diluted to appropriate concentrations in complete medium. In both cases, the final DMSO concentration did not exceed 0.25%. Having obtained an optimum DMSO concentration and cell density, cells were prepared and then treated with resveratrol (0.39, 0.78, 1.56, 3.13, 6.25, 12.5, and 25 μg/mL) and A2L (12.5, 25, 50, 100, 200, 400, and 800 μg/mL). The treated cells were incubated for 72 h with the effects of treatment being monitored at 24 h-intervals. At each interval, the plates were stained and then imaged with an ImageXpress MicroXL high-content fluorescence microscope.

### Mitochondrial toxicity testing

The Mitochondrial ToxGlo™ Assay used here is a cell-based assay that employs multiplexed chemistry for predicting potential mitochondrial dysfunction as a result of xenobiotic exposure. The assay is based on the differential measurement of biomarkers associated with changes in cell membrane integrity and cellular ATP levels relative to vehicle-treated control cells. Ten microlitres (10 μL) of culture medium containing approximately 1500 cells is plated on opaque-walled, black tissue culture plates compatible with fluorescent and luminescent measurements (clear bottoms). Being attachment-dependent, cells were incubated for 24 h to adhere to the substrate of the plate. The cells were then treated with 10 μL of sample and incubated for 24 h. Using an electronic multichannel pipette, 5 μL of 5X Cytotoxicity Reagent was dispensed to each well of the plate and mixed briefly (1 min) by orbital shaking (1300 rpm) to ensure Reagent/sample homogeneity and to mitigate possible Reagent/sample partitioning. The plate was then incubated at 37 °C for 30 mins and fluorescence was measured with rhodamine 110 filter set at 485 nm_Ex_/525 nm_Em_ using Flexstation 3 microplate reader (Molecular Devices, Sunnyvale, CA). Thereafter, the assay plate was equilibrated to room temperature (10 mins) and 25 μL of ATP Detection Reagent was added to each well. The plate was mixed by orbital shaking (1300 rpm) for 3 mins and luminescence was measured by using Wallac microplate reader.

### Caspase 3/7-activity

Caspase 3/7-activity was assessed with Caspase-Glo 3/7 luminescent Assay reagents. Cells were plated at 1500 per well in a volume of 12.5 μL culture medium in white 384-multiwell plates and allowed to attach for 24 h. After treatments (vehicle or test substances) in a volume of 12.5 μL culture medium, plates were subsequently incubated for 24 h. Then 25 μL of Caspase-Glo 3/7 Reagent was added to each well containing 25 μL of blank, negative control cells or treated cells in culture medium. Contents of wells were mixed using a plate shaker at 1300–1500 rpm for 30 s and the plates were incubated at room temperature for 1 h before measuring luminescence on Analyst GT multimode reader (Molecular Devices Corporation, Sunnyvale, CA, USA).

### Determination of adenosine triphosphate (ATP) levels

ATP levels were measured using CellTiter-Glo Luminescent Cell Viability Assay. Cells were plated at 3000 per well in triplicate in white 384-multiwell plates and allowed to attach for 24 h. After treatments (vehicle or test substances) in a volume of 12.5 μL culture medium, the plates were subsequently incubated for 24 h and then a volume of CellTiter-Glo reagent equal to the content of each well was added. After 10 mins, luminescence was read using Wallac plate reader (Perkin-Elmer, USA), and signal intensity was calculated relative to in-plate DMSO control wells.

### Determination of glutathione levels (antioxidant status)

Reduced-oxidised glutathione levels were determined by using the luminescence-based GSH/GSSG-Glo™ Assay reagents. Cells were plated at 3000 per well in a volume of 25 μL culture medium in white 384-multiwell plates and allowed to attach for 24 h. After treatments (vehicle or test substances) in a volume of 25 μL culture medium, plates were subsequently incubated for 24 h, cell culture medium/treatment was removed and plates were treated according to manufacturer’s instructions to determine the levels of total and oxidised glutathione. After 15 mins of incubation to stabilise the signal, luminescence was read using Wallac plate reader (Perkin-Elmer, USA). GSH/GSSG ratios are calculated directly from relative luminescent unit (RLU) measurements.

### Statistical analysis

For statistical analysis, data were analysed using SigmaPlot 11.0 and GraphPad Prism 5. The results were expressed as mean ± SD (standard deviation) and the median inhibitory/cytotoxic concentration (I/CC_50_) values were obtained from the linear regression plots. One-way analysis of variance (ANOVA) was used to assess differences between means (student’s t test, where applicable); if significant differences were found (*p* < 0.05, 0.01, or 0.001), Tukey-Kramer multiple comparison test was considered for post-test.

## Results

### Quantitative phytochemical analysis shows the significantly higher content of phenolics than flavonoids in A2L

A comparison of the total phenolic and flavonoid contents based on gallic acid and quercetin as the standard compounds, respectively indicates that A2L is highly rich in phenolic contents (605.6075 ± 0.6182 μg GAE/mg extract) as compared with its flavonoid contents (119.4792 ± 3.1302 μg QE/mg extract).

### Resveratrol displays mitogenic activity while A2L shows antiproliferative activity

**Fig. 1:**
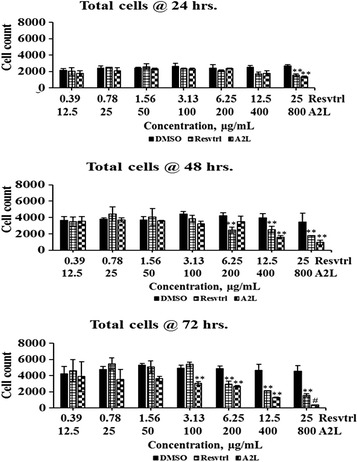
Determination of total cells. Numerical changes associated with treatment of HepG2 cells with resveratrol (Resveratrol) or extract (A2L) followed up for three days. *p < 0.05; **p < 0.01; ^#^ *p* < 0.001

Assessment of the proliferative activities of resveratrol and A2L in HepG2 cells for 72 h shows, on one hand, that resveratrol is mitogenic at lower concentrations throughout the period of treatment. On the other hand, A2L is consistent in its antiproliferative activity. For both resveratrol and A2L, cell proliferation seems not remarkably affected at 24 h after treatment. Overall, both resveratrol and A2L are antiproliferative at high concentrations being more pronounced with A2L which significantly (*p* < 0.05; 0.01; 0.001) displays a time- and dose-dependent antiproliferative activity. As expected, the negative control (DMSO-treated cells) maintained a similar proliferation pattern at the various concentrations within the same time of exposure (Fig. 1).

### Resveratrol and A2L could not induce the death of HepG2 cells at lower concentrations

**Fig. 2:**
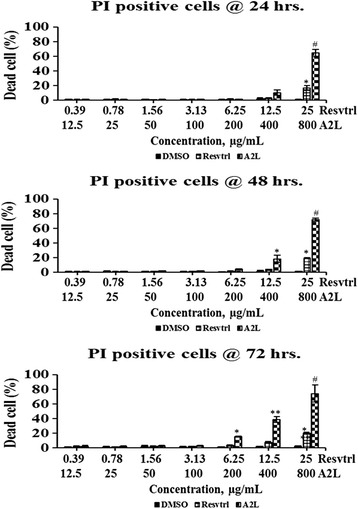
Quantification of treatment-induced cell death. Concentration- and time-dependent assessment of cytotoxicity resulting from treatment of HepG2 cells with resveratrol or A2L followed up for three days. *p < 0.05; **p < 0.01; ^#^ *p* < 0.001

Assessment of percentage dead cell based on the number of cells that are positive for propidium iodide indicates that resveratrol and A2L could not significantly (*p* > 0.05) induce HepG2 cell death at lower concentrations for the 72 h of exposure. One sharp difference that can be noticed is that A2L shows higher tendencies to be death-inducing in a time-dependent manner than resveratrol. At the highest concentration, A2L maintains a significantly (*p* < 0.001) strong death-inducing ability from 24 h through 72 h of exposure (Fig. 2).

### Total, positive, and negative nuclear sizes are affected in a time- and concentration-dependent manner

**Fig. 3:**
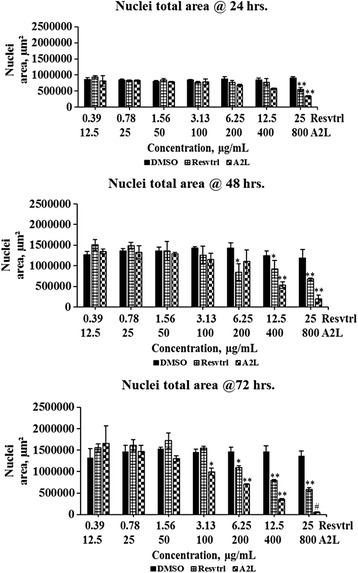
Determination of nuclei total area. Changes in total nuclear size associated with treatment of HepG2 cells with resveratrol or A2L followed up for three days. *p < 0.05; **p < 0.01; ^#^ *p* < 0.001

**Fig. 4:**
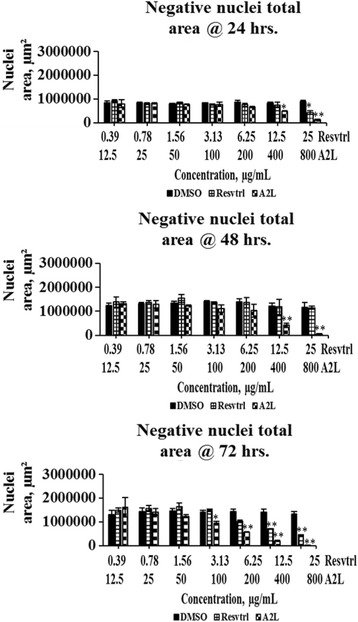
Determination of negative nuclei total area. Changes in negative nuclear size associated with treatment of HepG2 cells with resveratrol or A2L followed up for three days. *p < 0.05; **p < 0.01

**Fig. 5:**
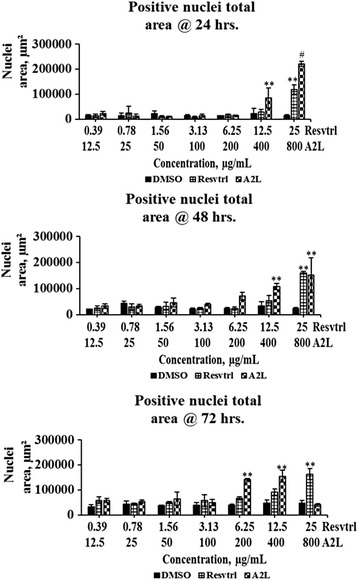
Determination of positive nuclei total area. Changes in positive nuclear size associated with treatment of HepG2 cells with resveratrol or A2L followed up for three days

The total nuclear area (Fig. 3) which indicates the overall combination of the area occupied by the genetic materials of both dead and live cells, followed the same trend with cell count (Fig. 1). In the case of the negative nuclei total area, a parameter that measures the genetic/nuclear size of live cells, it shows no changes at lower concentrations but a significant (*p* < 0.01) increase at higher concentrations most especially at 72 h post-treatment (Fig. 4). Lastly, the positive nuclei total area, which indicates the nuclear/genetic size of dead cells, shows a gradual increase in a time- and dose-dependent manner (Fig. 5).

### Both resveratrol and A2L do not possess mitotoxic potential

**Fig. 6:**
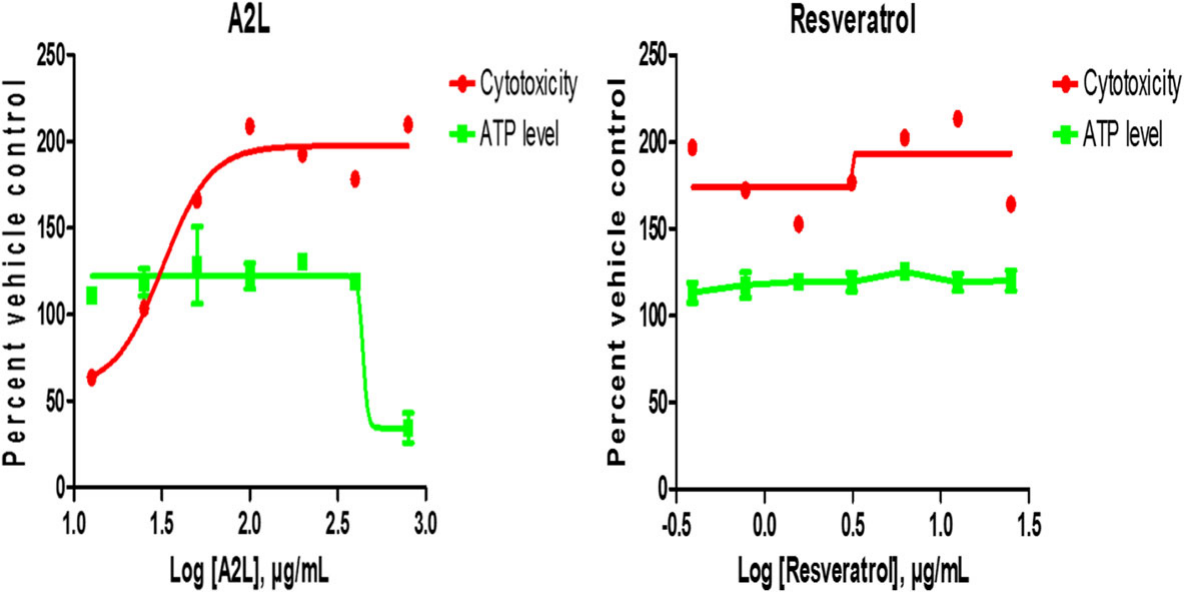
Assessment of mitochondrial toxicity. Changes in the levels of cytotoxicity/membrane integrity (MI) and ATP determine if a treatment is mitotoxic

Assessment of the influence of both resveratrol and A2L on possible mitotoxicity shows that both are unable to induce a depletion in the levels of ATP across the various concentrations of treatment considered in this study. The only exception being A2L which significantly depleted the ATP levels at its highest concentration (Fig. 6).

### HepG2 cell death induced by both resveratrol and A2L involves the activation of executioner caspases

**Fig. 7:**
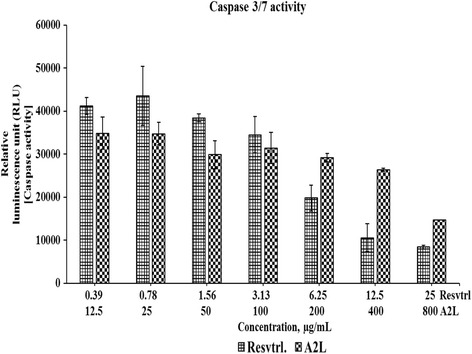
Caspase activation levels. This shows the trend of caspase activities induced by treatment with either resveratrol or A2L

Across the various concentrations of treatment, resveratrol and A2L demonstrated the induction of the apoptosis-inducing caspases 3 and 7. For both treatments, activation of these caspases are more pronounced at the lower concentrations compare to the higher concentrations. As the concentrations increase, resveratrol shows a more drastic reduction in the levels of these caspases as compared with the minimal change recorded for A2L (Fig. 7).

### Depletion in ATP levels seems to account principally for the cell death induced by A2L

**Fig. 8:**
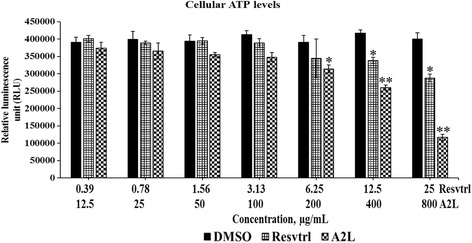
Effects of treatment on ATP levels. Variations in ATP status of HepG2 cells treated with resveratrol or A2L. *p < 0.05; **p < 0.01

Both resveratrol and A2L could not significantly (*p* > 0.05) affect the ATP levels at lower concentrations, but A2L significantly (*p* < 0.05) exhibited ATP-depletion capacity at higher concentrations (Fig. 8).

### Resveratrol and A2L are antioxidant boosters for HepG2 cells

**Fig. 9:**
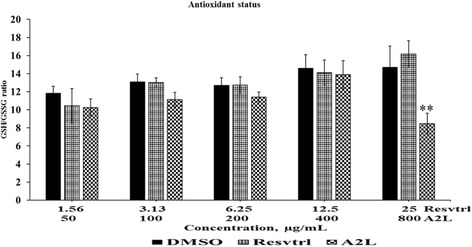
Effects of treatment on anti-oxidant status. Anti-oxidant status (GSH/GSSG ratio) of HepG2 cells treated with resveratrol or A2L. **p < 0.01

The antioxidant status of HepG2 cells was unaffected significantly (*P* > 0.05) by resveratrol and A2L as compared with the negative control. The only exception being that A2L demonstrated higher reducing potential than resveratrol throughout all the concentrations of treatment ultimately culminating in a significant (*p* < 0.01) decrease in the antioxidant status at the highest concentration of A2L (Fig. 9).

### Resveratrol and A2L induced morphological changes in HepG2 cells

**Fig. 10:**
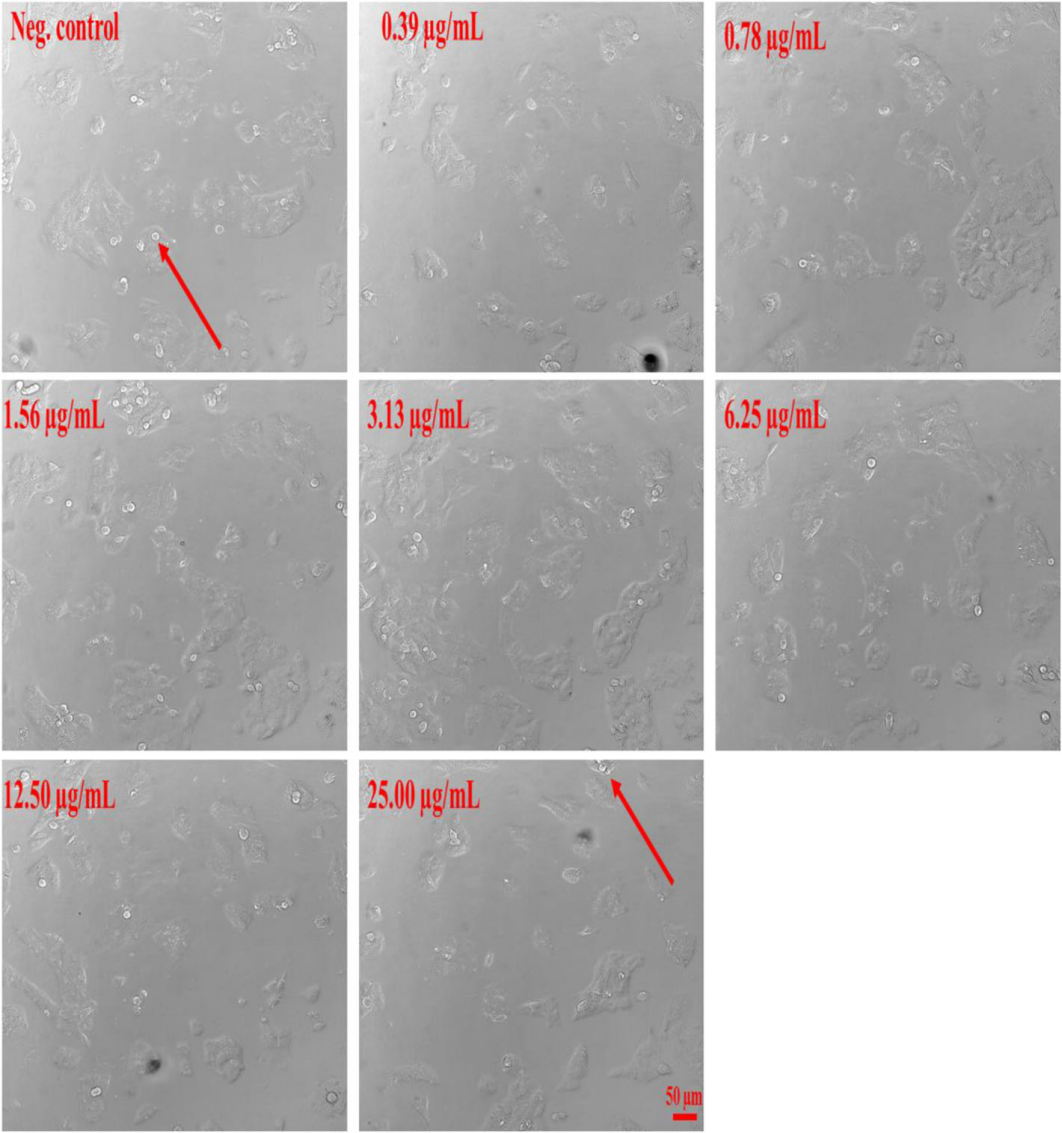
Changes in cellular morphology as recorded using a widefield fluorescent microscopy. Resveratrol-induced morphological changes in HepG2 cells after 24 h of treatment (×20)

**Fig. 11:**
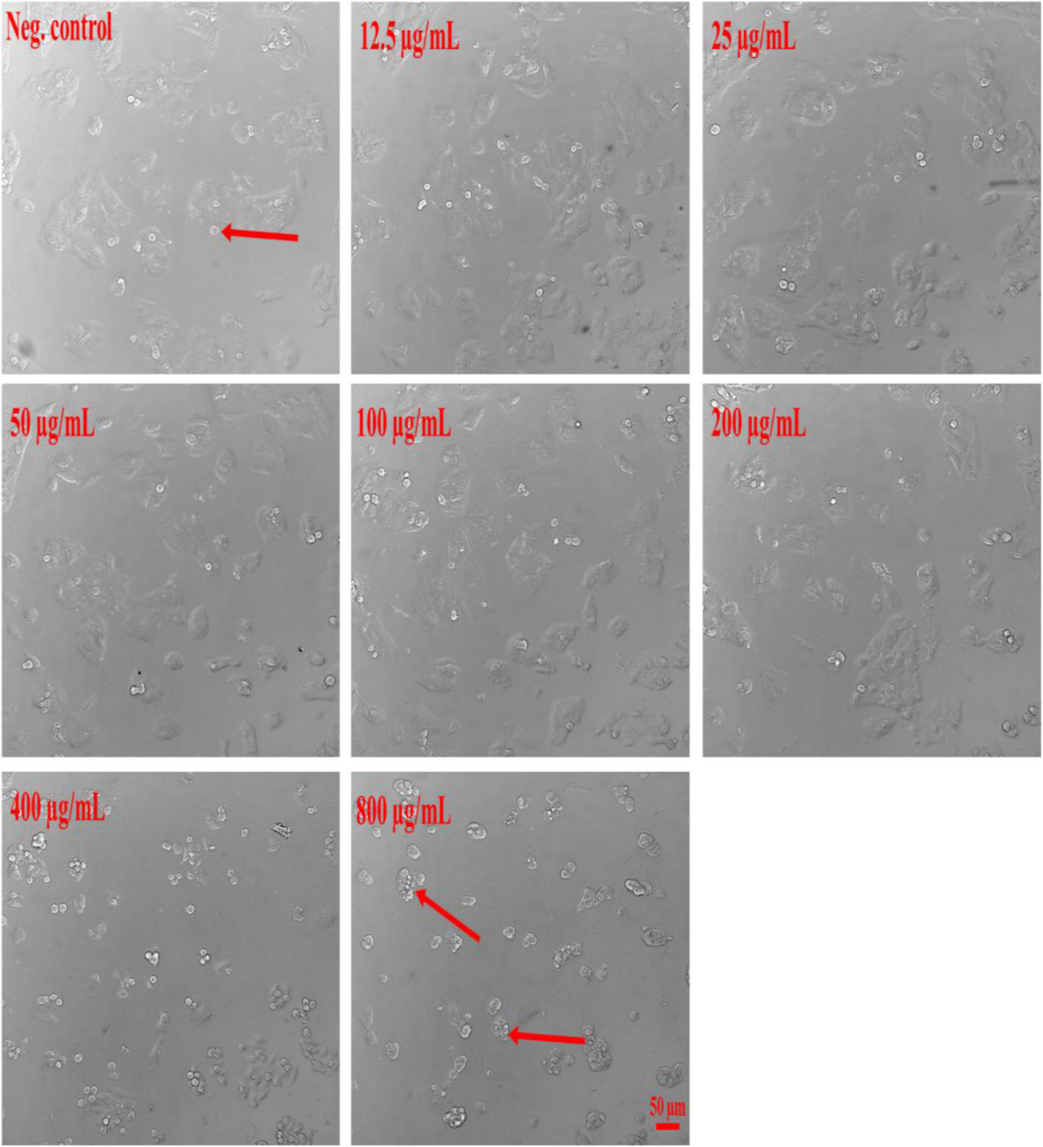
Changes in HepG2 cell morphology as recorded using a widefield fluorescent microscopy. A2L-induced morphological changes in HepG2 cells after 24 h of treatment (×20)

Morphological assessment of HepG2 cells shows a more pronounced effect induced on the cells by A2L most especially at its highest concentration as compared with resveratrol (Figs. 10 and 11).

## Discussion

The high morbidity and mortality rates associated with hepatocellular carcinoma (HCC) has been attributed principally to its chemoresistance with proven resistance to chemotherapeutic agents, such as adriamycin (ADM), cisplatin, 5-fluorouracil (5-FU) and doxorubicin [13, 14]. It is known that HCC is a major public health problem in many parts of the world, with high incident areas in East Asia and sub-Saharan Africa [15]. Our efforts in this work were channeled towards application of a plant-derived natural product in the management of HCC.

Various reports have shown that free radicals/reactive oxygen species (ROS), such as nitric oxide, hydroxyl radical, superoxide anion, are generated from both endogenous and exogenous sources. These products are generally reactive with cellular macromolecules, such as lipids, proteins, deoxyribonucleic acid (DNA). resulting in lipid peroxidation, degradation/denaturation of proteins, oxidation of DNA, respectively. These are the molecular basis of many diseases including cancer such as HCC [16, 17]. Findings from the present study demonstrated that the extract (A2L) used in this work possesses a very strong free radical scavenging activity as compared with ascorbic acid, the reference compound. This observed activity of A2L may be due to its high total phenolic content demonstrated in the quantitative phytochemical analysis. Undoubtedly, the flavonoid content, although quite minimal, may also be a contributing factor. Plant phenolics and flavonoids have been reported to exhibit efficient radical scavenging, metal chelating and antioxidant activities [18, 19].

The antiproliferative activity of resveratrol in HepG2 cells has been previously reported and here we confirm the same order except that its lower concentrations displayed mitogenic tendencies throughout the 72 h of exposure to treatment. In like manner, A2L almost demonstrated the same trend as resveratrol except that it exhibited greater antiproliferative activity in a time- and concentration-dependent manner [20, 21]. The values obtained for total nuclear, positive nuclear, and negative nuclear areas all corroborated the antiproliferative, apoptotic and necrotic effects of treatments depending on the concentrations of either resveratrol or A2L. Antiproliferative activity is indicated by the decrease in total and negative nuclei areas, while the increase in positive nuclei area implies necrotic cell-death [22–24].

In order to gain insights into the antiproliferative activities of both resveratrol and A2L, we assessed mitochondrial function and found that ATP synthesis was not significantly (*p* > 0.05) affected most especially at the lower concentrations, but became pronounced at higher concentrations. This is in line with a previous study [25]. Previous reports have indicated that resveratrol can induce mitochondrial biogenesis in HepG2 cells and may clearly explain the results we obtained in this present study [26, 27]. Although, this activity seems to be concentration-dependent as, at higher concentrations, both resveratrol and A2L were able to significantly (*p* < 0.05) reduce the ATP levels with A2L being potent (*p* < 0.01). The resultant effect of this disruption in ATP levels is the cell death induced at these concentrations.

In accordance with earlier reports, we observed that resveratrol maintained the antioxidant status of HepG2 cells throughout the concentrations considered in this study. This activity has been reported to be due to the capability of resveratrol to induce an increase in the activities of catalase (CAT), superoxide dismutase (SOD), GSH peroxidase (GPx), NADPH quinone oxidoreductase, and GSH-S-transferase with an attendant increase in intracellular glutathione (GSH) level [28, 29]. Results recorded on A2L indicated that the extract induced similar effect with resveratrol on the antioxidant status of HepG2 cells, with the exception of the highest concentration in which the extract significantly depleted the antioxidant level of the cells.

Resveratrol has been noted to induce apoptosis in cancer cells as it has been demonstrated in this study by the upregulation of the activities of effector caspases-3 and -7. For resveratrol-treated HepG2 cells, the trend of caspase activities obtained here across the various concentrations implies that the level of apoptotic cell death attained a threshold point after which other forms of cell death, most probably necrosis, becomes predominant. This point is evident from the decline in caspase activity. As for the A2L-treated cells, apoptotic level seems to have been well-maintained except at the highest concentration in which there was a remarkable reduction in the caspase activities [8, 30].

## Conclusion

Our findings validated the antiproliferative activities of resveratrol in HepG2 hepatocellular carcinoma cells. Here, for the first time we have been able to demonstrate the significant time- and concentration-dependent antiproliferative capability of the ethanol leaf extract of *A. leiocarpus* (A2L)*.* However, both resveratrol and the extract are non-cytotoxic except at high concentrations in which A2L is significantly more cytotoxic on HepG2 liver cancer cells than resveratrol in a time-dependent manner. Mechanisms of cell death by both treatments seem not to be ATP- or antioxidant-depletion dependent.

## Abbreviations

A2L: ethanol leaf extract of *Anogeissus leiocarpus*;
ATP: adenosine triphosphate
CAT: catalase
DMEM: Dulbecco’s Modified Eagle’s Medium
DMSO: dimethyl sulphoxide
DNA: deoxyribonucleic acid
GAE: gallic acid equivalents
GPx: glutathione peroxidase.
GSH: reduced glutathione
HCC: hepatocellular carcinoma
I/CC_50_: median inhibitory/cytotoxic concentration
QE: quercetin equivalents
ROS: reactive oxygen species
SD: standard deviation
SOD: superoxide dismutase
